# Prevalence of type 2 diabetes and impaired fasting glucose in patients affected by rheumatoid arthritis: Results from a cross-sectional study: Erratum

**DOI:** 10.1097/MD.0000000000008132

**Published:** 2017-09-15

In the article, “Prevalence of type 2 diabetes and impaired fasting glucose in patients affected by rheumatoid arthritis: Results from a cross-sectional study”,^[1]^ which appeared in Volume 96, Issue 34 of *Medicine*, the titles for Tables 3 and 4 should have appeared as below.

Table 3: Regression analyses of predictors of T2D

Table 4: Regression analyses of predictors of IFG
